# Evaluating the association of rs6983267 polymorphism of the *CCAT2* gene with thyroid cancer susceptibility in the Iranian Azeri population

**DOI:** 10.22099/mbrc.2023.47622.1839

**Published:** 2023

**Authors:** Masoud Jabraili, Solmaz Moniri-Javadhesari, Nasser Pouladi, Mohammad Ali Hosseinpour-Feizi

**Affiliations:** 1 Department of Cellular and Molecular Biology, Faculty of Basic Sciences, Azarbaijan Shahid Madani University, Tabriz, Iran; 2 Department of Biology, Faculty of Natural Sciences, University of Tabriz, Tabriz, Iran

**Keywords:** Thyroid cancer, Polymorphism, rs6983267, lncRNA, CCAT2

## Abstract

Thyroid cancer is the most common malignancy of the endocrine system. LncRNAs play critical role in various cellular processes and are associated with several diseases. CCAT2 is a lncRNA molecule overexpressed in thyroid cancer. Single nucleotide polymorphisms in* CCAT2 *gene can cause changes in the structure and function of CCAT2 transcripts and susceptibility to several diseases. This study aimed to evaluate the association of rs6983267 in *CCAT2 *gene with thyroid cancer susceptibility in the Azeri population of Iran. In this "case-control" study, genomic DNA was extracted from peripheral blood of 102 individuals affected by thyroid cancer and 103 healthy individuals as controls. Genotyping was performed using TETRA-ARMS polymerase chain reaction. Statistical analysis showed no significant association between genotypes and/or alleles with the occurrence of thyroid cancer in the studied population, patients' gender, and tumor type. Nevertheless, we found that the allelic and genotypic distribution of this SNP was associated with the size of thyroid tumors in patients. It is assumed that investigating more individuals from both case and control group may further determine the genotypic and allelic frequencies of this SNP locus in Iranian-Azeri population.

## INTRODUCTION

Thyroid cancer (TC) is the eighth most common cancer worldwide and the most common endocrine malignancy [1]. The prevalence of TC has been increasing over the last few decades [2]. Like other malignancies, the development of thyroid tumors is a multi-step process involving various genetic and epigenetic changes. Cancer genomics studies revealed variable degrees of genetic alterations including; duplications, inversions, rearrangements, and single nucleotide polymorphisms in TC [3, 4]. Single nucleotide polymorphisms (SNP) are the most common genetic variation in the human genome and considered for about 90% of genome variations [5, 6]. SNP is a change of a single nucleotide at a specific position in the genome and the frequency of an SNP must be at least one percent (>1%) of the population [7].

SNPs can be located in different regions of the genome [8]. SNPs within the genes encoding long non-coding RNAs (lncRNAs) can affect the secondary structure and function of lncRNAs [9]. LncRNAs are a group of non-coding RNAs (ncRNAs) with a length of more than 200 nucleotides [10]. They are structurally similar to mRNAs, and are produced through DNA transcription[11]. LncRNAs play a role in various cellular and molecular processes and pathogenic processes (such as tumor formation and progression) [12-14].

CCAT2 (Colon Cancer Associated Transcript 2) is a lncRNA molecule involved in the occurrence and progression of thyroid tumors. This lncRNA molecule transcribed from a gene with the same name and was first discovered in colon cancer [15-17]. The rs6983267 is a bi-allelic SNP in the chromosomal region 8q24.21, the same chromosomal region where the gene encoding the CCAT2 lncRNA molecule is also located; this gene is transcribed into about 1.7 kilo bases CCAT2 lncRNA and can have one of two types of nucleotides G or T at base pair 662, the same part corresponding to rs6983267. Nucleotide substitution in this SNP locus causes a change in the secondary structure of the CCAT2 lncRNA [4, 15].

As a result, investigating the relationship between the allelic status of this SNP in the *CCAT2* gene and susceptibility to various malignancies, including TC, can be very important. In this regard, this study was conducted to investigate the relationship between the allelic and genotypic distribution of rs6983267 with susceptibility to thyroid cancer and with some pathobiological characteristics of patients, in a population belonging to the northwest of Iran.

## MATERIALS AND METHODS

In this case-control study, 102 individuals affected by thyroid tumors and 103 healthy individuals with no familial history of cancer or other thyroid diseases were participated. All the participants belonged to the Azeri population from the northwest region of Iran. Informed consent was obtained from all participants in the study and all procedures were approved by the Ethics Committee of Azerbaijan Shahid Madani University (IR.AZARUNIV.REC.1401.020). Table 1 summarized characteristics of the studied population.

**Table 1:** Demographic characteristics of the studied population

**Parameter**	**Case no (%)**	**Control no (%)**
**Gender**		
** Female**	72 (70.5%)	73 (70.9%)
** Male**	26 (25.5%)	30 (29.1%)
**Unknown**	4 (4.0%)	0 (0%)
**Total**	102 (100%)	103 (100%)
		
**Age**		
**39.2±13.5**	102 (100%)	103 (100%)
		
**Tumor type**		
** FTC**	24 (25.0%)	
** PTC**	62 (64.6%)	
** Other types**	10 (10.4%)	
** Total**	96 (100%)	
		
**Tumor size**		
** ≤2 cm**	26 (38.8%)	
** >2 cm**	41 (61.2%)	
** Total**	67 (100%)	

**DNA** **Extraction and Genotyping:** Genomic DNA was extracted from 2 ml of peripheral blood of each case and control participants by salting-out method. Tetra ARMS-PCR was performed using two pairs of primers: non allele-specific outer forward (5′-TTT TTA GCA GCT GCA TCG CTC-3′) and outer reverse (5′-AGG AAA CTG AAC TGT GGG GTT G-3′) primers, T allele-specific inner forward (5′-CTT TGA GCT CAG CAG ATG AAG GT-3′) and G allele-specific inner reverse (5′-AAA ATT CTT TGT ACT TTT CTC AGC GC-3′) primers. The primers were designed using Gene Runner version 6.5 software, PRIMER1: primer design for Tetra-primer ARMS-PCR online tool (at: http://primer1.soton.ac.uk/primer1.html) and Primer-BLAST. 

After the initial denaturation (at 95°C for 5 min), touchdown method was used in the first 5 cycles including denaturation (at 95°C for 30 s), annealing (at 59-56°C for 30 s), extension (at 72 °C for 30 s). After 30 cycles, the reaction was terminated by final extension (at 72°C for 7 min). PCR products were electrophoresed on 3% agarose gel. After gel electrophoresis, three bands with lengths of 437 bp (base pair), 267 bp, and 219 bp were appeared. The 437 bp band should be present in all samples and is considered as the PCR positive control. Also, the 267 bp and 219 bp bands represented G and T alleles, respectively (see Fig. S1).

To ensure the accuracy of genotyping results, one sample from each genotypic pattern, was sequenced by the Sanger method (Fig. S2). The obtained results were aligned with the sequences in GenBank and confirmed the results obtained by TETRA-ARMS PCR.

**Statistical analysis:** The significance of the data was evaluated using the chi-square test ($x^{2}$). Also, the odds ratio (OR) was calculated for the genotypes (using binary logistic regression models) in three genetic models: dominant, recessive, and co-dominant. IBM SPSS Statistics 26 software was used for this purpose. Also, in cases where the frequency of variables was less than 5, Fisher's exact test was performed. In all the statistical tests, a significance level of less than 0.05 has been considered. Also, in the calculations related to the odds ratio, a 95% confidence interval (CI) was considered.

## RESULTS

Genotypic distribution was consistent with the Hardy-Weinberg equilibrium in both control ($x^{2}$=1.50, df=1, P=0.219) and case ($x^{2}$= 3.16, df=1, P=0.075) groups. Also, it was found that the genotypes determined by Tetra ARMS-PCR method are in full agreement with the results of Sanger sequencing. There was a difference in genotypic and allelic frequency of rs6982367 between the control and case groups. However, this difference is not statistically significant (Table 2). Examination of the genotypic distribution of the rs6982367 in dominant, recessive, and co-dominant genetic models, also did not show significant difference between the control and case individuals.

**Table 2:** Genotypic and allelic distribution of rs6983267 SNP in case and control individuals

**Genotypes/alleles**	**Case no (%)**	**Control no (%)**	**OR**	**95% CI**	**P**
**GG**	31 (30.4%)	25 (24.3%)	1.0	-	-
**GT**	42 (41.2%)	45 (43.7%)	1.329	0.677-2.607	0.409
**TT**	29 (28.4%)	33 (32.0%)	1.189	0.826-1.707	0.352
**G**	104 (51.0%)	95 (46.1%)	1.0	-	-
**T**	100 (49.0%)	111 (53.9%)	1.215	0.825-1.791	0.325

The relationship between the genotypic and allelic distribution of the rs6983267 with pathobiological characteristics of patients, including patients’ gender, tumor type, and tumor size was also investigated. No significant relationship was found between the allelic or genotypic distribution of rs6982367 and the gender of the patients as well as the FTC (follicular thyroid carcinoma) and PTC (papillary thyroid carcinoma) tumor types.

Nevertheless, it was found that there is a significant relationship between the genotypic and allelic distribution of rs6982367 and tumor size (P<0.05); In this way, the frequency of GG and GT genotypes in patients with tumors >2 cm is higher than in patients with tumors ≤2 cm, while the frequency of TT genotype in patients with tumors ≤2 cm is higher than in patients with tumors >2 cm. Also, the frequency of the G allele in patients with tumors >2 cm, compared to patients with tumors ≤2 cm, is higher than the frequency of the T allele, while in patients with tumors ≤2 cm, the frequency of the T allele is higher than the frequency of G allele (Table S1).

## DISCUSSION

SNPs are considered as potential cancer biomarkers that may lead to differences in disease susceptibility in different individuals, disease prognosis and outcome [18, 19]. SNPs can be valuable in early diagnosis and targeted treatment (personalized medicine) of cancers [20]. A large number of SNPs associated with diseases (including cancer) have been identified through GWAS studies [21]. An example of such SNPs is the rs6982367, which is located in the 8q24.21 chromosomal region in the gene encoding the CCAT2 lncRNA molecule, a lncRNA that is found to be involved in the occurrence of TC [4, 15, 16, 22].

The results of the present study indicated that there is no significant relationship between the allelic and genotypic distribution of rs6983267 SNP of the *CCAT2* gene and susceptibility to TC in the population belonging to the northwest of Iran. Also, no correlation was found between the allelic and genotypic distribution of this SNP with the gender of the patients and with the occurrence of FTC and PTS tumors in the mentioned population. Nevertheless, it was found that there is a correlation between the frequency of the G allele of rs6983267 SNP of the *CCAT2* gene and the presence of thyroid tumors larger than 2 cm ($x^{2}$=3.862, *df*=1, P=0.049).

## Acknowledgement:

The authors of this article Acknowledge all the individuals who participated in this research.

## Conflict of Interest:

The authors have no conflicts of interest to declare.

## Supplementary materials
