# Supplementary material for: Evaluating the association of rs6983267 polymorphism of the CCAT2 gene with thyroid cancer susceptibility in the Iranian Azeri population
Source: Mol Biol Res Commun. 2023;12(3):127–31. doi: 10.22099/mbrc.2023.47622.1839 (PMC10387174; doi:10.22099/mbrc.2023.47622.1839)
Supplement: Supplementary file 1 [file mbrc-12-127-s001.pdf]

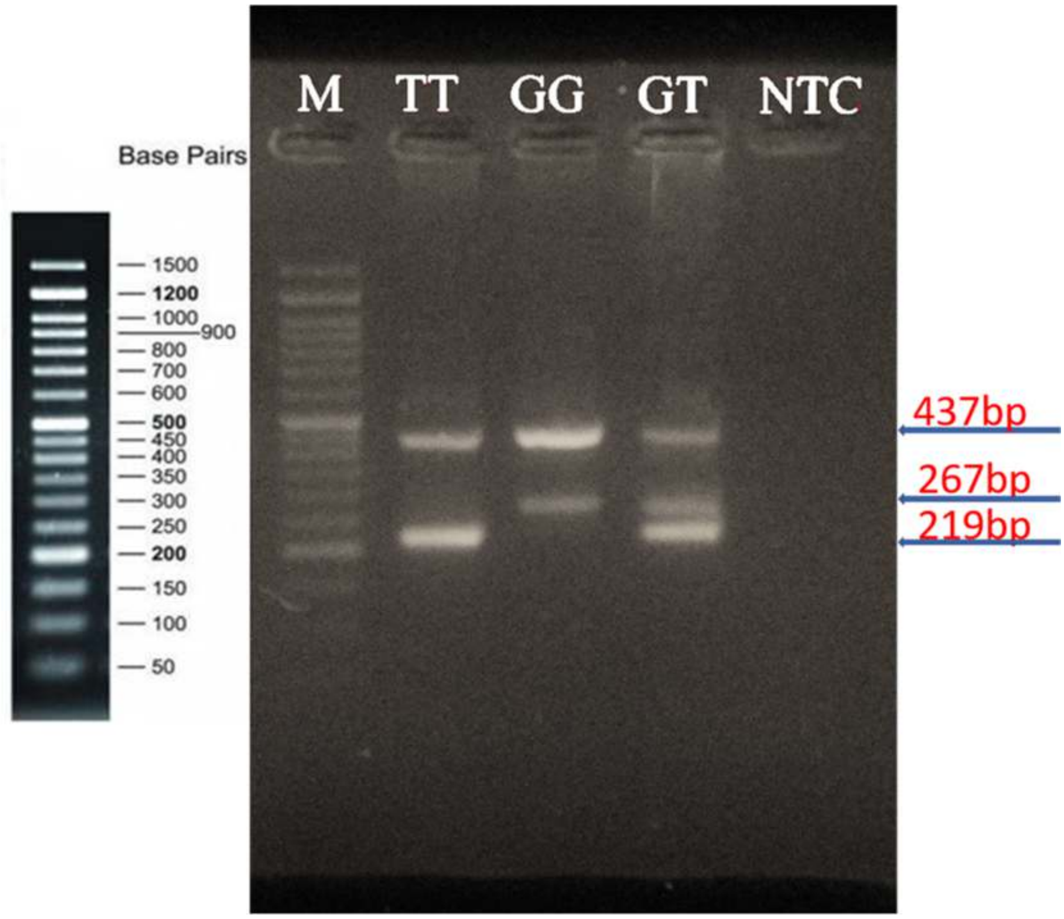

Figure S1: Gel electrophoresis and genotypic patterns (M: molecular marker, NTC: no template control)

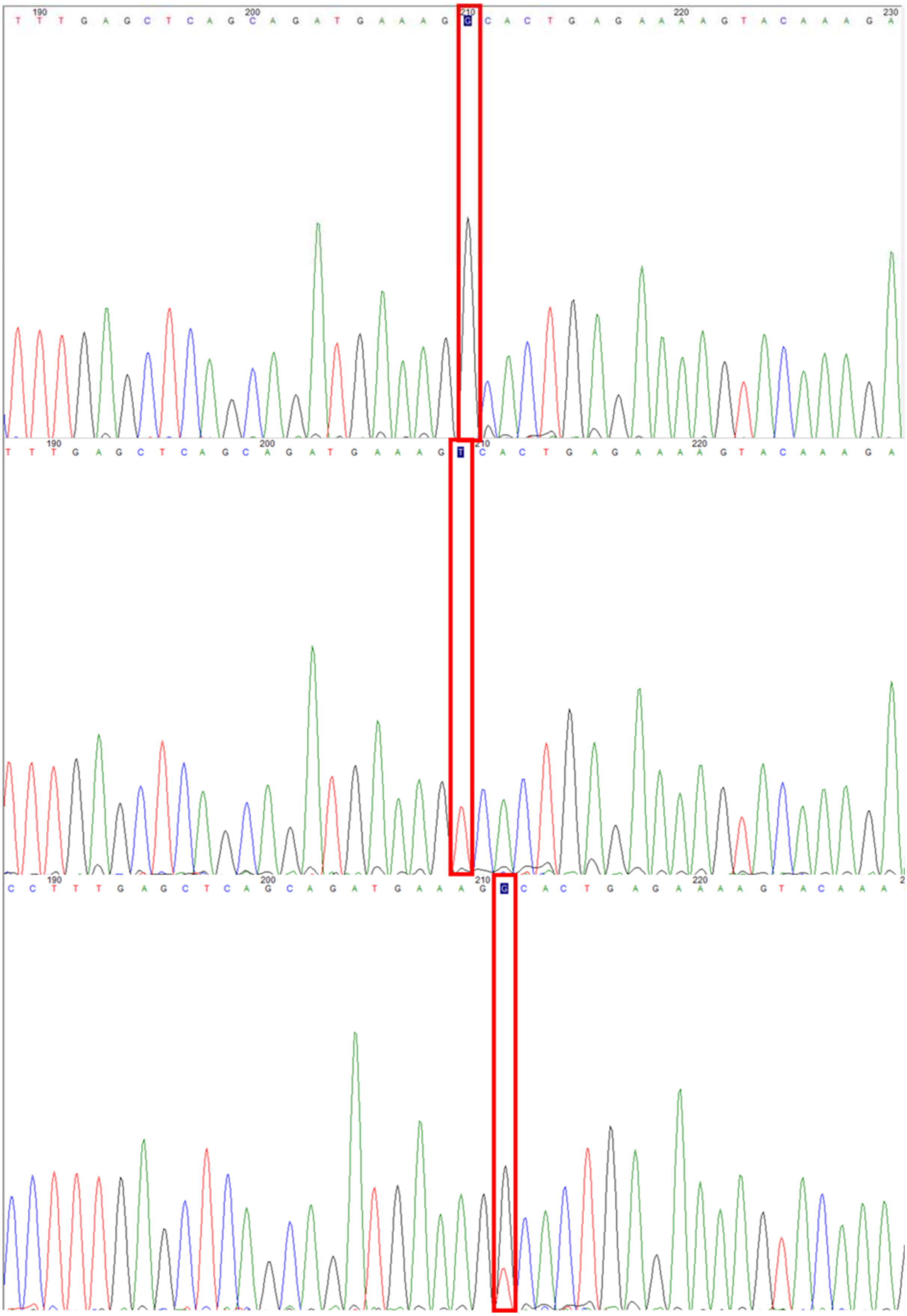

Figure S2: The results of sequencing by Sanger method (top: GG genotype, center: TT genotype, bottom: GT genotype)

Table S1: Allelic and genotypic distribution of rs6983267 SNP considering patients gender, tumor type, and tumor size

Parameter	Genotype/allele no (%)	X^2 (*df*)	P
**Gender**			
**Female**			
	GG 22 (30.6%)	1.812 (2)	0.422
	GT 27 (37.5%)		
	TT 23 (31.9%)		
	G 71 (49.3%)	0.639 (1)	0.424
	T 73 (50.7%)		
**Male**			
	GG 8 (30.8%)	1.812 (2)	0.422
	GT 13 (50.0%)		
	TT 5 (19.2%)		
	G 29 (55.8%)	0.639 (1)	0.424
	T 23 (44.2%)		
**Tumor types**			
**FTC**			
	GG 8 (33.3%)	2.497 (2)	0.333
	GT 12 (50.0%)		
	TT 4 (16.7%)		
	G 28 (58.3%)	1.849 (1)	0.174
	T 20 (41.7%)		
**PTC**			
	GG 17 (27.4%)	2.497 (2)	0.333
	GT 24 (38.7%)		
	TT 21 (33.9%)		
	G 58 (46.8%)	1.849 (1)	0.174
	T 66 (53.2%)		
**Tumor size**			
**≤2 cm**			
	GG 9 (34.6%)	11.108 (2)	0.003
	GT 6 (23.1%)		
	TT 11 (42.3%)		
	G 24 (46.2%)	3.862 (1)	0.049
	T 28 (53.8%)		
**>2 cm**			
	GG 15 (36.6%)	11.108 (2)	0.003
	GT 22 (53.6%)		
	TT 4 (9.8%)		
	G 52 (63.4%)	3.862 (1)	0.049
	T 30 (36.6%)		
